# Endodontic Surgery for Separated Instrument Removal: Success Rates and Techniques in a Systematic Review

**DOI:** 10.3390/dj13100449

**Published:** 2025-09-30

**Authors:** Mario Dioguardi, Ciro Guerra, Khrystyna Zhurakivska, Diego Sovereto, Lorenzo Lo Muzio, Angelo Martella, Andrea Ballini, Eleonora Lo Muzio, Stefania Cantore

**Affiliations:** 1 Department of Clinical and Experimental Medicine, University of Foggia, 71122 Foggia, Italy; ciro_guerra.556675@unifg.it (C.G.); khrystyna.zhurakivska@unifg.it (K.Z.); diego_sovereto.546709@unifg.it (D.S.); lorenzo.lomuzio@unifg.it (L.L.M.); 2 DataLab, Department of Engineering for Innovation, University of Salento, 73100 Lecce, Italy; angelo.martella@unisalento.it; 3 Department of Life Science, Health and Health Professions, Link Campus University, 00165 Rome, Italy; 4 Department of Biomedical and Neuromotor Sciences, University of Bologna, 40125 Bologna, Italy; eleonora.lomuzio@unibo.it; 5 Department of Precision Medicine, University of Campania Luigi Vanvitelli, 81100 Caserta, Italy; stefania.cantore@unicampania.it

**Keywords:** endodontic, endodontic surgery, fractured, retreatment, ultrasonic, separated, broken, failure endodontic, instrument endodontic

## Abstract

**Background:** Instrument separation is a frequent issue in root canals, often complicated by intriguing anatomical variations that make treatment more challenging. These variations in canal structure can lead to various iatrogenic complications, such as missed canals, instrument separation, gouging, perforation, and overextension of obturation materials. One such complication is instrument breakage, which can disrupt the cleaning and shaping processes and potentially cause pain or discomfort. **Materials and Methods:** The present systematic review was conducted following PRISMA guidelines and the Cochrane Handbook for Systematic Reviews of Interventions. The present systematic review aimed to identify all clinical trials focused on the removal of separated instruments from endodontic canals using an endodontic surgical approach. **Results:** A total of 21 studies were included, reporting 22 cases involving surgical approaches for separated instrument removal. **Conclusions:** The analysis of available evidence, although prim Information added.arily based on clinical cases and case series, emphasizes that surgical approaches for removing separated endodontic instruments are a viable therapeutic option when non-surgical treatments are ineffective or not feasible. Techniques such as apicoectomy, intentional replantation, surgical removal, and the technique provide innovative, customized solutions for addressing complications related to separated instruments, showing favorable clinical and radiographic success rates in follow-up assessments.

## 1. Introduction

The fracture of endodontic instruments within root canals, although infrequent in individual endodontic treatments, represents a significant issue when considering a growing number of treated teeth [1]. The fracture rate of instruments ranges from 2% to 8%, yet not all fractured parts necessarily remain within the canal [2]. Often, the separated portion is easily retrieved with predictable techniques, while in other cases, the fractured fragment may be bypassed and left within the root canal system, inadvertently becoming part of the filling material. In certain situations, however, removal is not feasible; the success rate of removing a fractured instrument declines sharply when it is located beyond the natural curvature or even at the root apex [3]. Under such conditions, the risk of root perforation increases. The most affected teeth are lower molars (accounting for 50% of cases), particularly in the mesial root [4].

Endodontic instrument fractures are attributed primarily to two mechanisms: cyclic fatigue and torsional fatigue [5]. Modern NiTi (Nickel-Titanium) endodontic instruments show a higher incidence of fracture (1.3–10%) compared to stainless steel instruments [6], despite their greater resistance [7]. This may be due to the fact that stainless steel instruments often display clinically visible distortions and deformations before breaking, prompting clinicians to discard the instrument at the onset of plastic deformation [8]. In contrast, NiTi instruments, both manual and rotary, may exhibit no visible signs of torsional or cyclic fatigue, making their reuse in additional canals more prone to unexpected breakage [9]. Generally, manual NiTi instruments are more challenging to remove than rotary ones due to their greater torsional engagement with canal walls [10].

The presence of fractured endodontic instruments in the canal system negatively impacts orthograde endodontic treatment, obstructing proper cleaning and shaping of the apical root canal, especially in teeth with periapical lesions. Therefore, instrument removal is always preferable; however, when removal is not possible, bypassing the fragment—incorporating it into the root canal filling material—may be a viable alternative [11]. In some cases, periapical surgical interventions may be employed.

The management of fractured instruments in root canals is a significant challenge in endodontics. Accurate assessment of the clinical scenario is essential to select the most appropriate approach, improving treatment prognosis and increasing long-term success rates [12].

Unlike previous reviews that combined surgical and non-surgical retrieval [2,13], this review focuses primarily on the surgical management of fractured endodontic instruments, detailing the available techniques (apical surgery, surgical removal, intentional replantation, “pushed” approach)—and summarizing the published clinical evidence. Attention is given to guided methods, as reported, including the use of CBCT and 3D-guided navigation. By collating these relatively under-reported yet clinically demanding scenarios, the review delineates current practice and highlights key evidence gaps to inform prospective registries and future comparative studies.

The systematic review questions posed by the authors are as follows: What evidence exists regarding the success of surgical endodontic approaches for removing retained fractured instruments? What are the real advantages of endodontic surgical techniques compared to non-surgical endodontic treatments aimed at removing or bypassing the instrument? What are the strategies for removing separated endodontic instruments that involve a surgical approach?

Therefore, the objective was to summarize the indications, techniques, and reported outcomes of surgical management of fractured endodontic instruments when orthograde retrieval or bypass are not feasible. This systematic review focuses descriptively on the surgical strategies adopted and on follow-ups. It does not attempt comparative and quantitative analyses of efficacy compared to nonsurgical management or between surgical techniques.

## 2. Materials and Methods

### 2.1. Protocol

The planning of the systematic review was implemented following the guidelines described in the Cochrane Handbook for Systematic Reviews of Interventions. The drafting of the review manuscript followed the recommendations of PRISMA (Preferred Reporting Items for Systematic Reviews and Meta-Analysis), and the protocol was registered on PROSPERO (International Prospective Register of Systematic Reviews) before carrying out the selection of articles and was registered with INPLASY2024110118 and DOI number 10.37766/inplasy2024.11.00118.

### 2.2. Eligibility Criteria

The research aimed to identify all clinical trials related to the removal of separated instruments within endodontic canals through an endodontic surgical approach.

The exclusion criteria encompassed all clinical studies that did not report data on the removal of endodontic instruments within the canals via a surgical approach, as well as studies that did not have an abstract in English. All literature reviews were excluded from the selection process.

Potentially eligible studies included all retrospective and prospective studies, case series, and trials that reported data on the surgical removal of separated instruments within the canals

The systematic review was performed by three reviewers (M.D., S.C., and A.B.) and followed the following steps:

Choice of reviewers (M.D. and S.C.) and a third reviewer (A.B.) as supervisor in case of conflict regarding the studies to be included, choice of outcomes to identify, choice of databases and keywords used, choice of eligibility criteria, choice of data to extract, and methods of synthesis and registration of the protocol on INPLASY [14].

Search and selection of studies through the use of databases with manual elimination or through the use of software (EndNote 8.0). This process was conducted independently, followed by comparing the selections made and deciding which studies to include.

Data extraction on tables performed independently (M.D. and S.C.), followed by data comparison to minimize the risk of errors in reporting information [15].

### 2.3. Sources of Information, Research, and Selection

The search for articles and reports was conducted by two reviewers using online search engines, who are also the authors of the manuscript (M.D. and S.C.). Preliminary exclusion criteria included linguistic restrictions: reports without at least an abstract in English were excluded using automated tools available in the databases. The search engines and databases utilized were PubMed, Scopus, and the Cochrane Library. Additionally, a grey literature search was conducted using Google Scholar, Science Direct, and Open Gray. To further minimize publication bias, the references of previous reviews on the removal of separated instruments were examined.

The following search terms were used on PubMed:

The records generated regarding “divorce” were excluded from the selection.

Search: (separated instrument endodontic) OR (broken endodontic) OR (apicoectomy) OR (apical surgery endodontic) OR (removal endodontic). Sort by: Most Recent;

((“divorce”[MeSH Terms] OR “divorce”[All Fields] OR “separated”[All Fields] OR “separation”[All Fields] OR “separations”[All Fields] OR “separabilities”[All Fields] OR “separability”[All Fields] OR “separable”[All Fields] OR “separate”[All Fields] OR “separately”[All Fields] OR “separates”[All Fields] OR “separating”[All Fields] OR “separational”[All Fields] OR “separative”[All Fields] OR “separator”[All Fields] OR “separators”[All Fields]) AND (“instrument”[All Fields] OR “instrument s”[All Fields] OR “instrumentation”[MeSH Subheading] OR “instrumentation”[All Fields] OR “instruments”[All Fields] OR “instrumented”[All Fields] OR “instrumenting”[All Fields]) AND (“endodontal”[All Fields] OR “endodontic”[All Fields] OR “endodontical”[All Fields] OR “endodontically”[All Fields] OR “endodontics”[MeSH Terms] OR “endodontics”[All Fields])) OR (“broken”[All Fields] AND (“endodontal”[All Fields] OR “endodontic”[All Fields] OR “endodontical”[All Fields] OR “endodontically”[All Fields] OR “endodontics”[MeSH Terms] OR “endodontics”[All Fields])) OR (“apicoectomy”[MeSH Terms] OR “apicoectomy”[All Fields] OR “apicoectomies”[All Fields]) OR ((“apical”[All Fields] OR “apically”[All Fields] OR “apicals”[All Fields] OR “apices”[All Fields]) AND (“surgery”[MeSH Subheading] OR “surgery”[All Fields] OR “surgical procedures, operative”[MeSH Terms] OR (“surgical”[All Fields] AND “procedures”[All Fields] AND “operative”[All Fields]) OR “operative surgical procedures”[All Fields] OR “general surgery”[MeSH Terms] OR (“general”[All Fields] AND “surgery”[All Fields]) OR “general surgery”[All Fields] OR “surgery s”[All Fields] OR “surgerys”[All Fields] OR “surgeries”[All Fields]) AND (“endodontal”[All Fields] OR “endodontic”[All Fields] OR “endodontical”[All Fields] OR “endodontically”[All Fields] OR “endodontics”[MeSH Terms] OR “endodontics”[All Fields])) OR ((“removability”[All Fields] OR “removal”[All Fields] OR “removals”[All Fields] OR “remove”[All Fields] OR “removed”[All Fields] OR “removement”[All Fields] OR “remover”[All Fields] OR “removers”[All Fields] OR “removes”[All Fields] OR “removing”[All Fields]) AND (“endodontal”[All Fields] OR “endodontic”[All Fields] OR “endodontical”[All Fields] OR “endodontically”[All Fields] OR “endodontics”[MeSH Terms] OR “endodontics”[All Fields])).

Translations separated: “divorce”[MeSH Terms] OR “divorce”[All Fields] OR “separated”[All Fields] OR “separation”[All Fields] OR “separations”[All Fields] OR “separabilities”[All Fields] OR “separability”[All Fields] OR “separable”[All Fields] OR “separate”[All Fields] OR “separately”[All Fields] OR “separates”[All Fields] OR “separating”[All Fields] OR “separational”[All Fields] OR “separative”[All Fields] OR “separator”[All Fields] OR “separators”[All Fields].

Instrument: “instrument”[All Fields] OR “instrument’s”[All Fields] OR “instrumentation”[Subheading] OR “instrumentation”[All Fields] OR “instruments”[All Fields] OR “instrumented”[All Fields] OR “instrumenting”[All Fields].

Endodontic: “endodontal”[All Fields] OR “endodontic”[All Fields] OR “endodontical”[All Fields] OR “endodontically”[All Fields] OR “endodontics”[MeSH Terms] OR “endodontics”[All Fields].

Apicoectomy: “apicoectomy”[MeSH Terms] OR “apicoectomy”[All Fields] OR “apicoectomies”[All Fields].

Apical: “apical”[All Fields] OR “apically”[All Fields] OR “apicals”[All Fields] OR “apices”[All Fields].

Surgery: “surgery”[Subheading] OR “surgery”[All Fields] OR “surgical procedures, operative”[MeSH Terms] OR (“surgical”[All Fields] AND “procedures”[All Fields] AND “operative”[All Fields]) OR “operative surgical procedures”[All Fields] OR “general surgery”[MeSH Terms] OR (“general”[All Fields] AND “surgery”[All Fields]) OR “general surgery”[All Fields] OR “surgery’s”[All Fields] OR “surgerys”[All Fields] OR “surgeries”[All Fields].

Removal: “removability”[All Fields] OR “removal”[All Fields] OR “removals”[All Fields] OR “remove”[All Fields] OR “removed”[All Fields] OR “removement”[All Fields] OR “remover”[All Fields] OR “removers”[All Fields] OR “removes”[All Fields] OR “removing”[All Fields].

On the Scopus and Cochrane Library platforms, a search was conducted in these two databases for the presence of the term (separated AND instrument AND endodontic) OR (broken AND endodontic) OR (apicoectomy) OR (apical AND surgery AND endodontic) OR (removal AND endodontic) in the title, abstract, and keywords (TITLE-ABS-KEY).

The keywords used on Google Scholar and ScienceDirect were “fractured separated endodontic”.

The literature search was completed on 23 July 2024, and a last update of the records was performed on 10 March 2025.

The data to be extracted included the study’s first author, date of publication, country where the research was conducted, number of teeth involved in the study, type of tooth or root, surgical technique employed, type of fractured instrument, patient information, and follow-up details.

### 2.4. Risk of Bias, Summary Measures, and Summary of Results

The risk of bias was assessed using a tool specific to case reports: the JBI Critical Appraisal Checklist for Case Reports [16].

The extracted results were organized into tables, incorporating data on the separated instruments and apicectomies. The potential to aggregate the data into summative numerical values was also considered.

### 2.5. Software and Tools

All data handling, statistical testing, and figure generation were performed using Python (version 3.10), employing the following libraries: Pandas (v. 1.5.3) for data manipulation and tabular analysis. Matplotlib (v. 3.7.1) for the generation of static figures, including bar charts, stacked bar plots, and horizontal bar plots. SciPy (v. 1.10.1) for statistical computations, including the chi-square test of independence.

## 3. Results

### 3.1. Selection of Studies

The following research question guided the study selections: What is the actual evidence of success for the surgical endodontic approach to removing separated instruments retained within root canals? What are the methods for removing separated instruments that involve a surgical approach?

**Figure 1:**
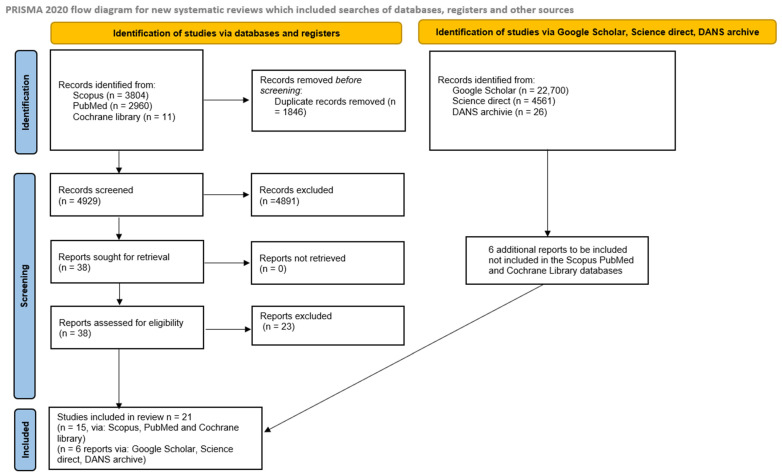
Flowchart of the article selection process.

The research phase involved the consultation and extraction of references from three databases: SCOPUS (3804), PubMed (2960), and a registry in the Cochrane Library (11), for a total of 6675 articles. No filters were applied in Scopus and PubMed to exclude studies published before a specific date or within a particular area of interest. Subsequently, references from Scopus and PubMed were imported into EndNote X8, and duplicates were removed both through the software and manually (1846 duplicates removed). A total of 38 studies were identified as potentially eligible, while 15 were included after full-text reading of the manuscripts. A further search of the grey literature (Google Scholar, Open Gray, and Science Direct) and previous systematic reviews identified six additional manuscripts for inclusion in this systematic review (Heydari et al. [17], Fujii et al. [18], Gandevivala et al. [19], Shenoy et al. [20], Nayak et al. [21], Noorani et al. [22]) (Figure 1).

Records were independently screened by two authors (M.D. and S.C.), and any ambiguous cases were resolved at the end of the selection process by involving a third author (A.B.) to address potential conflicts.

### 3.2. Data Characteristics and Data Extraction

The articles included in the review are as follows: Supraja et al. [23], Shekhawat et al. [24], Satheesh et al. [25], Heydari et al. [17], Kahler [26], Sudha et al. [27], Kadoo et al. [28], Wang et al. [29], Tariq et al. [30], Javed et al. [31], Coaguila-Llerena et al. [32], Fujii et al. [18], Sukegawa et al. [33], Gandevivala et al. [19], Shenoy et al. [20], Park et al. [34], Nayak et al. [21], Sheethi et al. [35], Pradeep et al. [36], Basatwar et al. [37], Noorani et al. [22].

The extracted data are presented in a table (Table 1), which includes first author, country of the study, total number of teeth, date of publication, country where the research was conducted, type of access cavity performed, number of teeth involved in the study, type of teeth, surgical technique employed, type of fractured instrument, patient information, follow-up details, and the teeth and roots involved.

The types of studies included were predominantly case reports, with three case series: Park et al. [34], Wang et al. [29], and Sudha et al. [27]. However, only in Sudha et al. [27] were multiple cases (*n* = 2) of surgical approaches to endodontic instrument removal described. A total of 22 teeth and 21 patients were treated across the studies, comprising 17 women and 5 men, aged between 13 and 65 years, with an average age of approximately 43 years. Follow-up was documented in 17 out of 22 case reports, with a range from 3 months to 2 years. The type, location, and number of teeth by type were as follows: 14 mandibular teeth, 2 anterior teeth (canines and incisors), and 3 premolars. Specifically: four 4.6, four 3.6, four 1.6, three 4.7, and one each of 1.2, 1.4, 4.4, 4.5, 1.3, 1.7, and 3.7.

The type of separated instruments was described in 11 cases, but was quite heterogeneous. In 8 cases, the instruments were presumed to have a consistent taper of 2% (e.g., endodontic files or H files), and in 2 cases, Protaper instruments were used. In 13 cases, the instrument’s location was documented as the apical third of the root. At least four types of treatment strategies were described: 8 cases using replantation, cases with surgical removal, 3 with apical surgery, and 2 with the pushed technique.

Furthermore, given the nature of the data, it was decided not to aggregate them through a meta-analysis.

### 3.3. Relationship Between Tooth Type and Surgical Technique

To explore whether the surgical approach varies by dental anatomy, tooth group (incisors/canines, premolars, molars; derived from FDI tooth numbers) was cross-tabulated against the technique employed (apical surgery, replantation, pushed, surgical removal). The distribution was non-uniform: replantation (8/8) and apical surgery (4/4) occurred exclusively in molars, whereas the pushed technique (2/2) was reported only in non-molars (one incisor/canine and one premolar). Surgical removal spanned all groups (five molars, two premolars, one incisor/canine).

A chi-square test of independence (excluding the “unknown” tooth group) yielded χ^2^ = 11.78, df = 6, *p* = 0.067. However, 10/12 expected cell counts were <5, contravening test assumptions; accordingly, the finding should be interpreted exploratorily rather than as confirmatory evidence of association. The observed and expected frequencies are reported in Table 2.

Tooth groups were derived from FDI numbering (1–3 = incisors/canines; 4–5 = premolars; 6–8 = molars). Given sparse case-report data and small expected counts, inferential testing is underpowered and descriptive patterns are emphasized.

**Figure 2:**
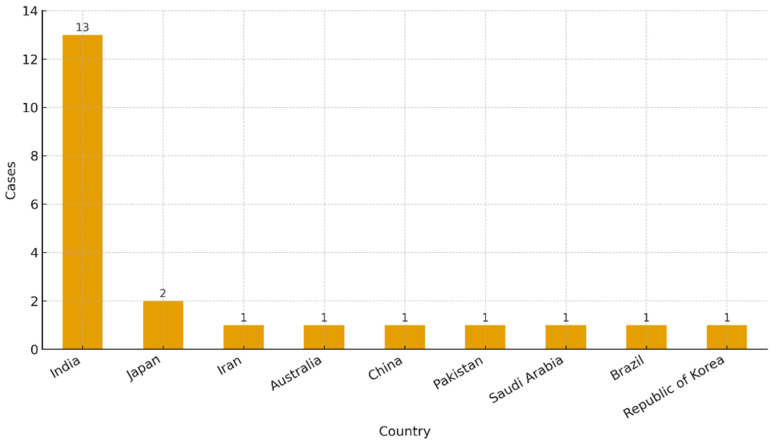
Distribution of clinical cases by country of origin.

**Figure 3:**
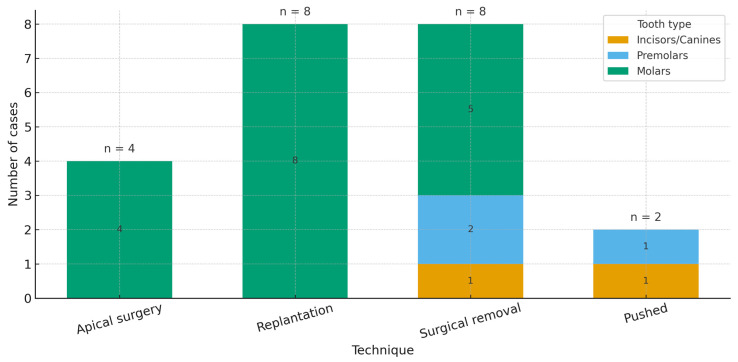
Distribution of cases according to surgical technique.

**Figure 4:**
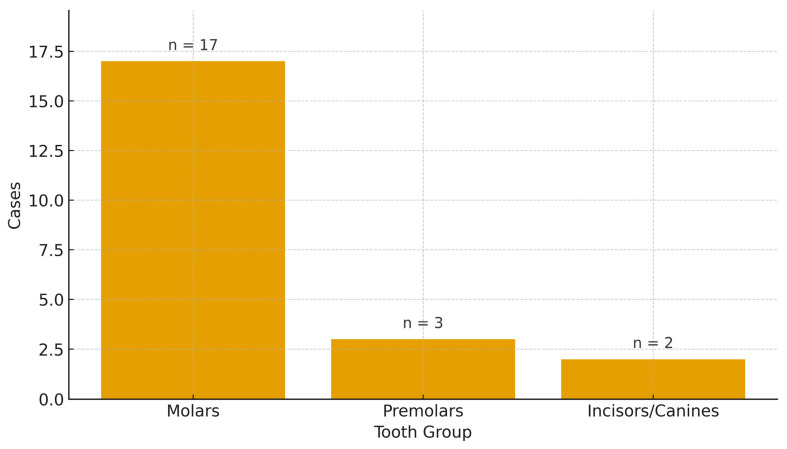
Distribution of involved teeth by anatomical group.

Across the updated case set (*n* = 22 cases from ≈20 studies after splitting multi-case reports), most cases originated from India (*n* = 13), with single-digit contributions from Japan (2), and one case each from Iran, Australia, China, Pakistan, Saudi Arabia, Brazil, and the Republic of Korea. Regarding techniques, surgical removal (*n* = 8) and replantation (*n* = 8) predominated, followed by apical surgery (*n* = 4) and pushed (*n* = 2). By anatomy, molars accounted for the majority (*n* = 17), with fewer premolars (*n* = 3) and incisors/canines (*n* = 2) (Figure 2, Figure 3 and Figure 4).

For apicoectomy, only four studies were included, and it was decided to summarize the main data in a single table. Table 3 summarizes the technical parameters for apicoectomy at the case level, including tooth involved, osteotomy size, use of the operating microscope, imaging modality, retrograde filling material, and length of apical resection (/ = not reported). In the extracted cases, MTA was consistently used for retrograde sealing; osteotomy diameters ranged from ~2 to 6 mm, apical resection ranged from ~3 to 8 mm, magnification was reported in most cases, and imaging included periapical radiography (RVG) and, where available, CBCT.

### 3.4. Analysis of Follow-Up Duration by Surgical Technique

**Figure 5:**
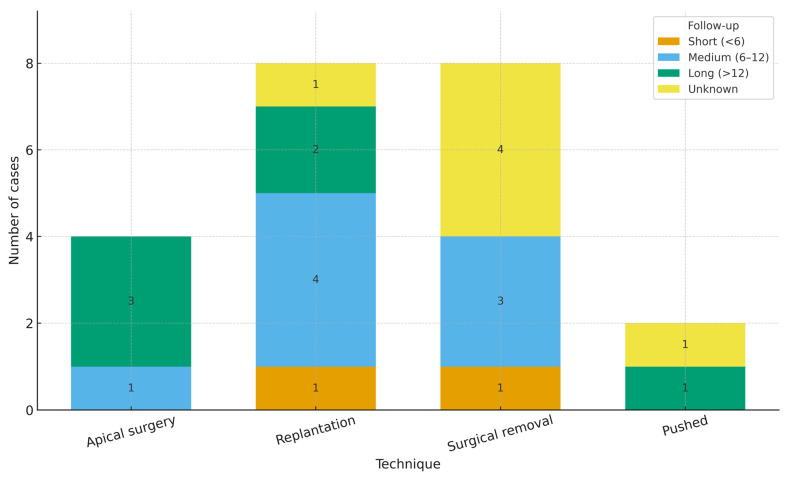
Analysis of follow-up duration by surgical technique. Stacked bars show the number of cases in each category (short <6 months; medium 6–12 months; long >12 months; unknown). Counts—apical surgery (*n* = 4): short 0, medium 1, long 3, unknown 0; replantation (*n* = 8): short 1, medium 4, long 2, unknown 1; surgical removal (*n* = 8): short 1, medium 3, unknown 4; pushed (*n* = 2): short 0, medium 0, long 1, unknown 1. The case with an ambiguous follow-up (“2 weeks or 1 year?”) was classified as unknown for the primary summary. These data are descriptive; small sample sizes preclude formal inference.

Follow-up periods were grouped a priori as short-term (<6 months), medium-term (6–12 months), long-term (>12 months), or unknown. A stacked bar chart displays the distribution across surgical techniques (Figure 5). The analysis reveals a heterogeneous pattern:

Surgical removal spans all categories but is dominated by short/medium observations with a high proportion of unknown durations (one short, three medium, zero long, four unknown; *n* = 8).

Replantation is predominantly medium-term (4/8), with additional long-term (2/8), short-term (1/8), and unknown (1/8) observations.

Apical surgery clusters toward longer observation (zero short, one medium, three long, zero unknown; *n* = 4).

Pushed techniques are rarely reported (*n = 2), with one long-term and one unknown follow-up. These distributions reflect reporting heterogeneity and small numbers; they should be interpreted descriptively rather than inferentially. The case with an ambiguous follow-up (“2 weeks or 1 year?”) was classified as unknown in the primary summary.

In three of four groups, unknown durations were still present, particularly among cases of surgical removal, highlighting the need for better standardization in reporting follow-up intervals in the case-based literature.

This distribution suggests that the choice of surgical technique is partially influenced by the anticipated need for clinical monitoring, which in turn may reflect the invasiveness, complexity, or healing risk associated with each procedure. These trends may inform both clinical decision-making and future study designs, where follow-up length should be tailored to the procedure’s biological and functional recovery expectations (Figure 5).

### 3.5. Risk of Bias

Given the types of studies included, the risk of bias was assessed using a tool specific to case reports: the JBI Critical Appraisal Checklist for Case Reports [16]. All included studies were evaluated using this scale. Some studies reported as case series contained descriptions of individual cases and were assessed accordingly for those individual cases. However, studies with a high risk of bias were excluded and are not shown in Table 4.

A qualitative assessment of the methodological quality of the included case reports was conducted using the JBI Critical Appraisal Checklist, which includes eight key criteria ranging from patient demographics to post-treatment outcomes and clinical significance. For each report, a “risk score” was calculated based on the number of questions with answers marked as “no” or “unclear”.

The results are summarized in a bar chart displaying only those studies with at least one criterion not adequately addressed (Risk Score ≥ 1). Three reports—Pradeep et al. [36], Sudha et al. (second case) [27], and Park et al. [34]—each presented three non-compliant criteria, suggesting a higher risk of bias and reduced reliability of the reported clinical course.

**Figure 6:**
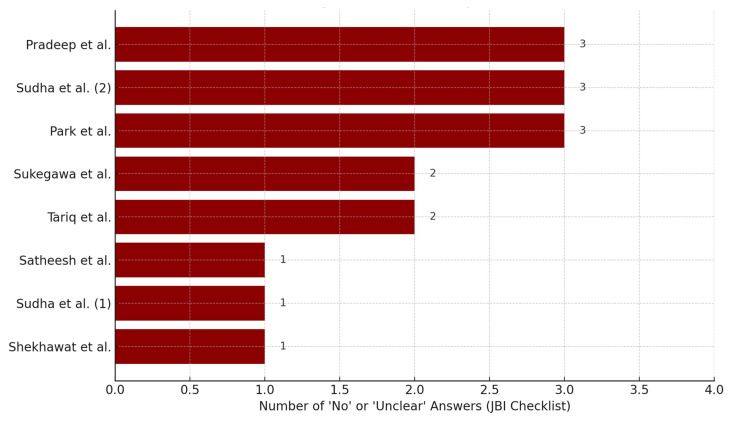
Studies with higher risk of bias (JBI—risk score ≥ 1). However, multiple cases (*n* = 2) of surgical approaches for endodontic instrument removal were described only by Sudha et al. [27], and reported as Sudha et al. (1) and Sudha et al. (2). Pradeep et al. [36], Park et al. [34], Sukegawa et al. [33], Tariq et al. [30], Satheesh et al. [25], Shekhawat et al. [24].

Other studies, such as those by Tariq et al. and Sukegawa et al., showed two unmet criteria, while a smaller number of reports (e.g., Satheesh et al. [25] and Shekhawat et al. [24]) exhibited only minimal concerns. Besides, multiple cases (*n* = 2) of surgical approaches for endodontic instrument removal were described only by Sudha et al. [27]. (Figure 6).

These findings highlight the heterogeneity in reporting quality among the available case reports. The presence of incomplete or ambiguous information—particularly regarding follow-up conditions or adverse events—may limit the ability to draw firm conclusions about the clinical success and reproducibility of the surgical approaches used for separated instrument removal.

## 4. Discussion

In this work, a systematic review of the literature was conducted to address the following questions: What are the actual success rates of the surgical endodontic approach for removing separated instruments retained within the root canals? What are the genuine advantages of surgical endodontic techniques compared to non-surgical approaches aimed at either removing or bypassing the separated instrument? Lastly, what strategies exist for the surgical removal of separated endodontic instruments?

From the initial stages of study selection and inclusion, it became evident that no clinical trials or retrospective studies specifically addressing surgical endodontics aimed at the removal of separated instruments retained within the canals were available. The literature predominantly comprised case reports and case series. Ultimately, 21 manuscripts were included, covering 22 surgical case reports, 12 of which were affiliated with institutions in India. The remaining studies were geographically diverse, with contributions from Iran, Japan, Pakistan, China, Australia, Saudi Arabia, Brazil, and South Korea. This distribution suggests a notable prevalence of interest in this topic within Asian countries, particularly India [38].

This may reflect a greater clinical interest in the issue of separated instrument fragments during endodontic procedures in these countries, particularly in India, where endodontics is an emerging specialty [39]. The field is experiencing advancements in techniques and tools, driven by a strong focus on developing innovative treatment methods and a clinical-scientific interest in optimizing outcomes and, above all, managing complications.

The ideal protocol for retrieving separated instruments aims to restore the case to its original state prior to the fracture, while preserving the tooth’s hard tissue and maintaining the integrity of its supporting structures [40].

As highlighted in the literature, surgical approaches to instrument removal must consider the position of the fractured instrument. If the instrument is located entirely beyond the apex, surgical removal becomes necessary, regardless of whether endodontic treatment or retreatment is planned. In other scenarios, intervention on the hard dental tissue is required, which may involve apical reshaping, intentional replantation, or, in some cases, pushing the separated instrument further into the canal to facilitate orthograde retrieval [41].

This systematic review is based entirely on case reports and small case series. Such designs preclude valid comparative inferences between surgical and non-surgical management, owing to the absence of control groups (reflecting the lack of randomized or otherwise controlled studies), the high risk of selection and publication bias, and heterogeneous, incompletely reported outcome definitions and follow-up. Accordingly, our synthesis should be interpreted as descriptive and hypothesis-generating, mapping the indications and technical variants of surgical strategies rather than establishing their superiority over non-surgical approaches, and it does not identify any one surgical technique as superior. Where head-to-head randomized trials are impractical, a realistic path forward is the development of prospective, multicenter registries with standardized outcome definitions and ≥12–24-month follow-up, and—where feasible—matched cohort designs to yield more informative comparative estimates.

In summary, the strategies described in the case reports can be categorized into four main approaches: apicoectomy [42], intentional replantation [43], surgical removal [32], and the “pushed” technique [33].

Each of these techniques will be analyzed in terms of its respective approaches, as well as its advantages and disadvantages.

### 4.1. Surgical Removal

This approach does not involve resection of the apical root but typically requires the creation of a surgical flap, followed by an osteotomy to access and remove the fractured instrument. An orthograde canal treatment may subsequently be performed, as indicated in the included studies [44].

Understanding the precise location of the fractured endodontic instrument is a critical aspect of this procedure. This can be partially achieved using 2D imaging, such as periapical radiographs or RVG (Radiovisiography)[45]. However, whenever possible, it should be supplemented with 3D imaging techniques like CBCT or CT, which provide invaluable information. These modalities not only help assess the extent of periapical lesions and the position of the instrument but also reveal its spatial relationship with critical anatomical landmarks [36].

Recovering a fractured fragment from the apical root of maxillary molars can be particularly challenging due to its proximity to the maxillary sinus. In such cases, removal may require elevation of the sinus membrane while accounting for and minimizing the risk of potential pinpoint membrane perforation [35].

Additionally, the proximity of the separated instrument to the mandibular canal poses significant surgical challenges, potentially leading to serious complications. A multicenter study conducted by Wessel and Gale [46] reported transient or permanent paresthesia of the lower lip in 20–21% of apicoectomy procedures on mandibular molars, with symptoms typically resolving within three months. However, permanent paresthesia was observed in approximately 1% of cases [46].

Rare and less-documented scenarios also exist, such as the case reported by Nayak et al. [21], where the fractured fragment was located within the mandibular foramen and associated with paresthesia. In this instance, a dermoaspiration technique was employed to retrieve the broken endodontic instrument, resulting in the resolution of dysesthesia symptoms. However, the tooth was subsequently extracted [21].

A thorough understanding of anatomical structures and their clinical implications—such as the maxillary sinus and the inferior alveolar canal—is essential for performing endodontic treatments and managing potential complications effectively.

To address these challenges, guided endodontic surgery, in conjunction with advanced radiographic imaging, can be utilized for retrieving separated instruments beyond the apex. This approach involves the use of a preformed guide created from data acquired via CBCT. Such techniques minimize the risk of damage to vital surrounding structures while ensuring accurate targeting and avoiding deviations into critical anatomical regions.

### 4.2. Pushed Technique

The pushed technique has been described in only two case reports: Heydari et al. [17] and Sukegawa et al. [33]. Although the pushed technique described by Heydari et al. [17] is not explicitly defined as surgical, it involves an unconventional removal approach. The technique described by the authors consists of an alternative method to remove a fractured instrument from the apical third of a maxillary lateral incisor via a fistulous tract. In this case, the instrument fragment, partially extended into the periapical lesion, was pushed back into the root canal (previously cleared of endodontic filling material from earlier treatments) using a long-shafted excavator. Access to the instrument was achieved through a pathological lesion, specifically a “fistula of the periapical lesion,” without employing a true surgical approach.

Interestingly, Elhakim et al. [47] describe how, following an acute suppurative apical periodontitis treated initially with orthograde drainage of pus, the separated instrument located in the periapical region gradually migrated into the root canal space during radiographic follow-ups. This allowed for a successful non-surgical removal of the fractured instrument [47].

Alternatively, Sukegawa et al. [33] used a fully surgical approach. Employing a three-dimensional navigation system, the surgeons localized the precise position of the fragment within the mandible. A small vestibular incision, approximately 15 mm, was made at the site closest to the fragment, followed by subperiosteal reflection. After creating a 3 mm bone window in the buccal cortical bone near the root apex, the fractured instrument was gently pushed from the apical region toward the crown and removed using mosquito forceps [33].

The advantages of these techniques can be summarized as reducing the need for extensive surgical interventions, minimizing invasiveness, and preserving surrounding tissues, while avoiding potential negative aesthetic effects associated with more destructive surgery. Furthermore, the surgical navigation system provides precise localization of the fragment within the bone or tooth, reducing the risk of damage to vital anatomical structures such as nerves and blood vessels. It also shortens the overall procedure time and facilitates faster patient recovery [48]. For example, the use of the pushed method through a fistulous pathway avoided surgical opening and demonstrated excellent healing in long-term follow-up [17].

Disadvantages include a steeper learning curve for mastering the guided navigation software and creating customized occlusal splints (a removable intra-oral appliance that covers the occluding surfaces to provide a temporary, balanced occlusion) [49]. Additionally, repositioning the fragment within the canal is not always guaranteed, particularly if the fragment moves to a position that is difficult to retrieve or bypass subsequently. In some cases, there is a risk of further damage to the canal structure. In summary, the disadvantages include the following: the need for specific expertise, longer preparation times, and potential complications in fragment repositioning [17].

Although this evidence is promising, it is important to note that it is predominantly based on two clinical case reports and has limitations compared to larger and controlled studies. Randomized trials with larger samples would be necessary to confirm and further quantify the success of these approaches conclusively.

### 4.3. Apicoectomy

Apicoectomy is required in cases where the fragment is retained in the apical third and cannot be removed using micro-surgical instruments such as needle holders and tweezers. Although apicoectomy is widely used in surgical endodontics, its application specifically for the removal of fractured instruments is poorly represented in the medical literature, with only three case reports available (Table 3), all performed on first molars [25,26,28].

The use of a surgical microscope is strongly recommended by the authors and has been documented in two out of three cases. The operating microscope allows for the vis [50] and accessory canals, which are often difficult to detect with traditional methods [51]. This enhances the ability to remove fractured instrument fragments and thoroughly clean the canals. The site preparation should include a minimal osteotomy (3–4 mm) to ensure better post-operative healing, and the apical resection can be performed using either rotary diamond burs or ultrasonic inserts [52].

The retrograde seal (root-end filling) after apical resection was completed in all cases using MTA, which offers good biocompatibility and bio-inductive properties, ensuring an effective seal [53]. The surgical site was closed with sutures.

Additionally, CBCT imaging during the diagnostic and preoperative phases aids in the accurate visualization of anatomical structures and the precise planning of the surgical procedure. Combined with digital impressions, this technology enables the creation and printing of virtual surgical models using 3D printers [50].

These techniques provide a highly effective and safe approach to managing cases of separated instruments retained within root canals, with documented clinical and radiographic success. In the case reports, radiographic follow-ups demonstrated complete healing of periapical lesions within 12–18 months, with no signs of recurrence or residual inflammation. Furthermore, these techniques are particularly effective in cases where fragments are located beyond the canal curvature or at the root apices, where orthograde treatment is difficult or impossible. Surgical intervention thus allows direct and secure access to the fragment for its removal.

Although endodontic surgical techniques deliver excellent outcomes in complex cases, they have some notable disadvantages. These include a higher operative risk in terms of complications, with an increased likelihood of anatomical damage, particularly near delicate structures such as the maxillary sinus or mandibular nerve. Additionally, while minimally invasive compared to traditional orthograde treatments, these procedures still require osteotomy and flap opening, which can result in postoperative discomfort and a longer recovery period than orthograde treatments.

Moreover, guided surgical methods, while highly precise, involve increased planning time and costs, which can extend the overall treatment duration compared to conventional endodontic approaches.

However, with the right skills and equipment, these challenges can be minimized to achieve optimal results.

### 4.4. Replantation

The removal of separated instruments can follow two pathways: non-surgical or surgical. It is also worth considering the possibility of retaining the instrument within the canal, which may be a deliberate choice if the endodontic treatment was performed on a vital, uncontaminated pulp and the fragment is located in the apical third.

A valid solution is represented by apicoectomy, although this approach has limitations, particularly when the tooth is in close proximity to critical anatomical structures, such as the thickness of the buccal cortical bone, the mandibular nerve, or the maxillary sinus [18]. In such contexts, intentional replantation may be considered. The American Association of Endodontists defines this procedure as “the insertion of a tooth into its alveolus after the tooth has been extracted for the purpose of performing treatment, such as root-end filling(s) or perforation repair.”

Several factors contribute to the success of replantation procedures, primarily organizational. The procedure must be swift, typically lasting no more than 10–15 min, and performed under sterile conditions. Splinting is another critical factor, with the element being stabilized for a period of 2 to 6 weeks. Failures are most commonly observed within the first year, which is why a follow-up of at least three years is recommended. Failures may manifest as root resorption, ankylosis, or replacement resorption, the latter being strongly influenced by the procedure’s duration, particularly the time the tooth spends outside the alveolus.

The presence of healthy cementum on the root surface is crucial in preventing ankylosis. Some authors suggest the use of tetracycline, citric acid, and ethylenediaminetetraacetic acid (EDTA) to create a root surface that promotes cellular adhesion and growth, thereby preventing ankylosis.

The extraction should be performed as atraumatically as possible to avoid damaging the periodontal ligament. Levers should not be used, and forceps should grip above the cementoenamel junction. Some clinicians immerse the extracted tooth intermittently in fresh cow’s milk, coat it with antibiotic paste, or use Hank’s Balanced Salt Solution (HBSS). The tooth should be kept moist with saline-soaked gauze. If the root or tooth fractures during extraction, the procedure must be stopped, and the tooth removed. Furthermore, the alveolus should be minimally manipulated, and any granulation tissue, blood clots, or debris should be removed through apical curettage [31].

After extraction, the apices and separated fragment are removed, usually with high-speed burs. Typically, about 3 mm of the apex should be removed [31]. The retrograde cavity should be prepared using ultrasonics and filled with a suitable retrograde filling material, such as MTA. Both the alveolus and roots should be irrigated with saline before replantation. The tooth is then repositioned, splinted, and occlusion is adjusted. Patients should be instructed not to chew hard foods on the reimplanted tooth [54].

Splinting remains a controversial aspect. Some recommend it only in cases of marked instability, while others advocate splinting in all cases to support periodontal tissues while allowing for minimal physiological movement [31].

Replantation is considered a simpler, less invasive, less expensive, and less time-consuming procedure compared to apicoectomy. It also appears to be more predictable in terms of risks such as resorption and ankylosis. The average retention time for reimplanted teeth ranges from 3 to 5 years [55]. Additionally, it offers the advantage of allowing complete visualization and treatment of otherwise inaccessible areas of the root [18].

While some authors believe that replantation should always be considered an alternative with reasonable chances of success, others view it as a last resort, to be undertaken only when other techniques have failed or are likely to fail. Replantation is not suitable for teeth with curved or flared roots, as these are at risk of fracturing during extraction, nor is it recommended for teeth serving as prosthetic abutments [55].

### 4.5. Limitations

This systematic review presents several limitations that should be acknowledged. Firstly, the majority of the included studies were single-case reports or small case series, which inherently carry a low level of evidence and a high risk of publication bias. The observational and retrospective nature of these reports limits the ability to establish causal relationships or generalize findings to broader populations.

Secondly, the heterogeneity across studies in terms of patient characteristics, anatomical sites, surgical approaches, and follow-up durations prevented the execution of a quantitative meta-analysis. Although an exploratory chi-square analysis was conducted to evaluate associations between surgical techniques and anatomical tooth types, this should be interpreted with caution due to the non-comparative nature of the source data.

Moreover, several studies lacked detailed reporting of key methodological domains, such as adverse events, structured follow-up outcomes, or standardized diagnostic protocols. This was confirmed by the risk of bias analysis using the JBI checklist, which revealed variable reporting quality across studies, with some presenting up to three non-compliant domains.

Finally, while graphical analyses were used to explore trends in country of origin, technique distribution, and follow-up duration, these are inherently descriptive and do not account for potential confounding factors.

Taken together, these limitations underscore the need for prospective studies with standardized methodologies and larger sample sizes to confirm the findings and improve the quality of evidence in the field of endodontic surgery for separated instrument removal.

Additionally, this review does not support claims of superiority of surgical versus non-surgical management for fractured endodontic instruments. Surgical options (apical surgery, targeted surgical removal, intentional reimplantation, and selected “push” techniques) remain viable in selected scenarios, particularly when orthograde retrieval or bypass are not feasible, but robust comparative conclusions require higher-level controlled evidence.

## 5. Conclusions

To minimize instrument separation and the accompanying stress and anxiety, proactive prevention should be the primary focus. In cases of separation, opting for safe retrieval or bypassing techniques is essential. The analysis of the available evidence, although limited to clinical cases and case series, highlights that the removal of separated endodontic instruments through surgical approaches represents a valid therapeutic option when non-surgical treatment proves ineffective or impractical. The techniques described, including apicoectomy, intentional replantation, surgical removal, and the “pushed” technique, offer innovative and tailored solutions to address complications arising from separated instruments, demonstrating good clinical and radiographic success rates in follow-ups.

While promising, many of these techniques are supported by limited studies. This underscores the need for further controlled clinical trials with larger sample sizes to validate and standardize these procedures.

The combination of advanced technologies, such as 3D printing for surgical models and CBCT imaging, with well-established surgical techniques, could represent a significant step forward in managing endodontic complications. However, it is essential to develop more standardized protocols and enhance operator training to ensure predictable outcomes and minimize complications.

## Figures and Tables

**Table 1:** Data extracted from the 20 studies included the following: \ data not available, ? unclear data, F female, M male; Tariq et al. [30]—unclear whether follow-up is at one year or two weeks.

Authors	Date	Nationality	Technique	Age/Sex Patient	Teeth, Root	Follow-Up	
**Supraja et al. [23]**	2023	India	Replantation	49, F	4.6, mesial third apical	6 months	Rotary NiTi file size 20, 6%
**Shekhawat et al. [24]**	2021	India	Replantation	13, M	3.6, mesial lingual third apical	1 year	File size 20, 2%
**Satheesh et al. [25]**	2017	India	Apical surgery	32, M	3.6, mesial buccal canal	18 months	\
**Heydari et al. [17]**	2016	Iran	Pushed	32, M	1.2, third apical	18 months	\
**Kahler [26]**	2011	Australia	Apical surgery	62, F	1.6, mesial buccal canal	12 months	Endodontic file
**Sudha et al. [27]**	2023	India	Surgery removal	35, F; 45, F	4.7, mesiobuccal; 1.4, buccal canal	6 months \	\
**Kadoo et al. [28]**	2024	India	Apical surgery	26, F	1.6, mesiobuccal root	24 months	\
**Wang et al. [29]**	2023	Chine	Replantation	45, F	4.6, mesiolingual root, third apical	3 months	M3 Pro-gold rotary files, United Dental Group, China
**Tariq et al. [30]**	2022	Pakistan	Replantation	34, M	3.6 mesiobuccal root, third apical	2 weeks, 1 year?	File size 25, 2%
**Javed et al. [31]**	2022	Saudi Arabia	Replantation	35, F	4.6, apical third of mesiobuccal canal	24 months	\
**Coaguila-Llerena et al. [32]**	2023	Brazil	Surgery removal	27, M	4.6, mesiobuccal cana l, third apical	12 months	Protaper S1
**Fujii et al. [18]**	2020	Japan	Replantation	30, F	1.6, distal root, third apical	12 months	\
**Sukegawa et al. [33]**	2017	Japan	Pushed	65, F	4.4 beyond the apex	\	\
**Gandevivala et al. [19]**	2014	India	Surgery removal	32, F	3.6 distal apical third	6 months	Protaper F2
**Shenoy et al. [20]**	2014	India	Replantation	37, F	3.7, mesiobuccal canal, apical third	1 year	25 K-file
**Park et al. [34]**	2012	Republic of Korea	Surgery removal	61, F	4.5	\	Endodontic file
**Nayak et al. [21]**	2011	India	Surgery removal	50, F	4.7	\	Endodontic file
**Sheethi et al. [35]**	2017	India	Surgery removal	45, F	1.7 mesiobuccal root apex	1 month	Endodontic file
**Pradeep et al. [36]**	2015	India	Surgery removal	32, F	1.3	\	H file
**Basatwar et al. [37]**	2022	India	Replantation	40, F	4.7, mesiobuccal and mesiolingual canal	2 years	\
**Noorani et al. [22]**	2017	India	Apical surgery	51, F	1.6 mesiobuccal root apex	2 years	\

**Table 2:** Chi-square analysis—surgical technique vs. tooth type; χ^2^ = 11.78, df = 6, *p* = 0.06702. Expected counts computed under independence; values <5 indicate sparse data and warrant caution in inference.

Chi-Square: 11.78, df: 6, *p*-Value: 0.06702			
Observed (Technique × Tooth Group)			
Technique	Incisors/Canines	Molars	Premolars
Apical surgery	0	4	0
Pushed	1	0	1
Replantation	0	8	0
Surgical removal	1	5	2
Expected frequencies (under independence)			
Apical surgery	0.36	3.09	0.55
Pushed	0.18	1.55	0.27
Replantation	0.73	6.18	1.09
Surgical removal	0.73	6.18	1.09

**Table 3:** Apicoectomy main data extracted, / data not reported.

First Autor	Teeth	Osteotomy	Microscopic	Imaging	Retrograde Filling Material	Resection
**Satheesh et al. [25]**	3.6	6 mm	no	RVG	MTA	8 mm
**Kahler [26]**	1.6	/	Yes	Rx periapical	MTA	3 mm
**Kadoo et al. [28]**	2.6	2–3 mm	yes	CBCT	MTA	/
**Noorani et al. [22]**	1.6	/	no	Rx periapical	/	/

**Table 4:** Risk of bias: JBI critical appraisal checklist for case reports, y (yes), n (no), u (unclear). Park et al. [34] case series (6 cases), only one case of fractured endodontic instruments, Gandevivala et al. [19] 2 case reports only one with surgical approach.

Author, Data	Were Patient’s Demographic Characteristics Clearly Described?	Was the Patient’s History Clearly Described and Presented as a Timeline?	Was the Current Clinical Condition of the Patient on Presentation Clearly Described?	Were Diagnostic Tests or Assessment Methods and the Results Clearly Described?	Was the Intervention(s) or Treatment Procedure(s) Clearly Described?	Was the Post-Intervention Clinical Condition Clearly Described?	Were Adverse Events (Harms) or Unanticipated Events Identified and Described?	Does the Case Report Provide Takeaway Lessons?
**Supraja et al. 2023 [23]**	y	Y	y	Y	y	y	y	y
**Shekhawat et al. [24]**	y	Y	u	Y	y	y	y	y
**Satheesh et al. [25]**	y	Y	u	y	y	y	y	y
**Heydari et al. [17]**	y	Y	y	y	y	y	y	y
**Kahler [26]**	y	Y	y	y	y	y	y	y
**Sudha et al. [27]**	u	Y	y	y	y	y	y	y
	u	Y	y	y	y	u	u	y
**Kadoo et al. [28]**	y	Y	y	y	y	y	y	y
**Wang et al. [29]**	y	Y	y	y	y	y	y	y
**Tariq et al. [30]**	y	Y	y	y	y	u	u	y
**Javed et al. [31]**	y	Y	y	y	y	y	y	y
**Coaguila-Llerena et al. [32]**	y	Y	y	y	y	y	y	y
**Fujii et al. [18]**	y	Y	y	y	y	y	y	y
**Sukegawa et al. [33]**	y	Y	y	y	y	u	u	y
**Gandevivala et al. [19]**	y	Y	y	y	y	y	y	y
**Shenoy et al. [20]**	y	Y	y	y	y	y	y	y
**Park et al. [34]**	u	Y	y	y	y	u	u	y
**Nayak et al. [21]**	y	Y	y	y	y	y	y	y
**Sheethi et al. [35]**	y	Y	y	y	y	y	y	y
**Pradeep et al. [36]**	u	Y	y	y	y	u	u	y
**Basatwar et al. [37]**	y	Y	y	y	y	y	y	y
**Noorani et al. [22]**	y	Y	y	y	y	y	y	y

## Data Availability

No new data.

## References

[1] Terauchi Y., Ali W.T., Abielhassan M.M. (2022). Present status and future directions: Removal of fractured instruments. Int. Endod. J..

[2] Dioguardi M., Dello Russo C., Scarano F., Esperouz F., Ballini A., Sovereto D., Alovisi M., Martella A., Lo Muzio L. (2024). Analysis of Endodontic Successes and Failures in the Removal of Fractured Endodontic Instruments during Retreatment: A Systematic Review, Meta-Analysis, and Trial Sequential Analysis. Healthcare.

[3] Suter B., Lussi A., Sequeira P. (2005). Probability of removing fractured instruments from root canals. Int. Endod. J..

[4] Vouzara T., Lyroudia K. (2018). Separated instrument in endodontics: Frequency, treatment and prognosis. Balk. J. Dent. Med..

[5] Setzer F.C., Böhme C.P. (2013). Influence of combined cyclic fatigue and torsional stress on the fracture point of nickel-titanium rotary instruments. J. Endod..

[6] Babu A. (2016). Comparative Assessment of Torque Expression of Stainless Steel, Nickel Titanium, Heat Activated Nickel Titanium and Titanium Molybdenum Alloy Wires—A Finite Element Method Study. Master’s Thesis.

[7] Feghali M., Xhajanka E., Di Nardo D., Bhandi S., Kassabian P., Seracchiani M., Gambarini G., Testarelli L. (2021). Incidence of different types of intracanal fracture of nickel–titanium rotary instruments: A systematic review. J. Contemp. Dent. Pract..

[8] Harrison T. (2020). Cyclic Fatigue Resistance of Nickel Titanium Rotary Files in the Martensitic State: A Systematic Review. Master’s Thesis.

[9] McGuigan M., Louca C., Duncan H. (2013). Endodontic instrument fracture: Causes and prevention. Br. Dent. J..

[10] Zanza A., D’Angelo M., Reda R., Gambarini G., Testarelli L., Di Nardo D. (2021). An update on nickel-titanium rotary instruments in endodontics: Mechanical characteristics, testing and future perspective—An overview. Bioengineering.

[11] Sira A., Nawar N.N., Saber S.M., Kim H.C. (2024). The effect of different separated file retrieval strategies on the biomechanical behavior of a mandibular molar: A finite element analysis study. J. Endod..

[12] Orozco-Ocampo Y.M., Escobar-Rincón D., Jiménez-García F.N., Álvarez-Vargas C.A., Jaramillo-Gil P.X. (2024). Factors influencing NiTi endodontic file separation: A thematic review. Dent. Med. Probl..

[13] Samiei M., Sabanik P., Avval S.T. (2025). Guided Endodontics for Non-surgical Root Canal Retreatment: A Systematic Review. Iran. Endod. J..

[14] Longo A., Zappatore M., Martella A., Rucco C. Enhancing Data Education with Datathons: An Experience with Open Data on Renewable Energy Systems. Proceedings of the 1st ACM SIGMOD International Workshop on Data Systems Education: Bridging Education Practice with Education Research, DataEd 2022.

[15] Caione A., Guido A.L., Martella A., Paiano R., Pandurino A. (2016). Knowledge base support for dynamic information system management. Inf. Syst. e-Bus. Manag..

[16] Ma L.L., Wang Y.Y., Yang Z.H., Huang D., Weng H., Zeng X.T. (2020). Methodological quality (risk of bias) assessment tools for primary and secondary medical studies: What are they and which is better?. Mil. Med. Res..

[17] Heydari A., Rahmani M., Heydari M. (2016). Removal of a Broken Instrument from a Tooth with Apical Periodontitis Using a Novel Approach. Iran. Endod. J..

[18] Fujii R., Morinaga K., Asai T., Aida N., Yamada M., Sako R., Furusawa M. (2020). Intentional Replantation to Treat Apical Periodontitis of Maxillary First Molar with Foreign Body Located Outside Apical Foramen Using CBCT: A Case Report. Bull. Tokyo Dent. Coll..

[19] Gandevivala A., Parekh B., Poplai G., Sayed A. (2014). Surgical removal of fractured endodontic instrument in the periapex of mandibular first molar. J. Int. Oral Health JIOH.

[20] Shenoy A., Mandava P., Bolla N., Vemuri S. (2014). A novel technique for removal of broken instrument from root canal in mandibular second molar. Indian J. Dent. Res. Off. Publ. Indian Soc. Dent. Res..

[21] Nayak R.N., Hiremath S., Shaikh S., Nayak A.R. (2011). Dysesthesia with pain due to a broken endodontic instrument lodged in the mandibular canal—A simple deroofing technique for its retrieval: Case report. Oral Surg. Oral Med. Oral Pathol. Oral Radiol. Endod..

[22] Noorani T., Ghani N.R.N.A., Asif J., Rahim I. (2017). Surgical endodontics to manage a separated instrument: A case report. Dent. Update.

[23] Supraja K.K., Sethupathi R., Diana D., Srinivasan M.R. (2023). Management of Separated Instrument at Apical Third of a Mandibular Molar by Intentional Replantation: A Case Report. J. Oper. Dent. Endod..

[24] Shekhawat D., Sharma N., Emmanuel B.J., Narwat S. (2021). Intentional replantation of a tooth with separated instrument: A case report. Int. J. Med. Dent. Case Rep..

[25] Satheesh S.L., Jain S., Bhuyan A.C., Devi L.S. (2017). Surgical Management of a Separated Endodontic Instrument using Second Generation Platelet Concentrate and Hydroxyapatite. J. Clin. Diagn. Res. JCDR.

[26] Kahler B. (2011). Microsurgical endodontic retreatment of a maxillary molar with a separated file: A case report. Aust. Dent. J..

[27] Sudha A., Krishnan A., Samant P.S., Dubey S. (2023). Guidodontics: A global positioning system (GPS) to surgical Endodontics—A case series. J. Conserv. Dent. JCD.

[28] Kadoo S., Patni P.M., Jain P., Agrawal N., Raghuwanshi S., Pandey S.H. (2024). An unusual case of maxillary first molar with seven canals and the successful surgical recovery of a separated instrument. J. Conserv. Dent. Endod..

[29] Wang Y., Hofmann M., Ruf S., Zhang J., Huang Q. (2023). Intentional replantation and dental autotransplantation of mandibular posterior teeth: Two case reports. Medicine.

[30] Tariq H., Ali M.B., Rizwan Z., Rizwan G., Leghari S., Ahmed A. (2022). Intentional re-implantation in a left mandibular second molar with broken file in apical third of mesiobuccal root: A case report. JPMA J. Pak. Med. Assoc..

[31] Javed M.Q., Zaman H., Srivastava S., Khan Z.J. (2022). Intentional Replantation of Mandibular First Molar with Two Years Follow Up- Case Report. J. Ayub Med. Coll. Abbottabad.

[32] Coaguila-Llerena H., Lazo-Quezada G., Teves A., Zevallos-Chávez M., Faria G. (2023). Removal of separated instruments from unfavourable locations: Case reports using the HBW ultrasonic ring or a surgical approach. Aust. Endod. J..

[33] Sukegawa S., Kanno T., Shibata A., Matsumoto K., Sukegawa-Takahashi Y., Sakaida K., Furuki Y. (2017). Use of an intraoperative navigation system for retrieving a broken dental instrument in the mandible: A case report. J. Med. Case Rep..

[34] Park S.S., Yang H.J., Lee U.L., Kwon M.S., Kim M.J., Lee J.H., Hwang S.J. (2012). The clinical application of the dental mini C-arm for the removal of broken instruments in soft and hard tissue in the oral and maxillofacial area. J. Cranio-Maxillo-Facial Surg. Off. Publ. Eur. Assoc. Cranio-Maxillo-Facial Surg..

[35] Sheethi K., Sheoran K., Thakur V.S. (2017). Surgical Removal of Fractured Endodontic Instrument Extending Beyond the Periapex of Mesiobuccal Root of Maxillary Second Molar through the Maxillary Sinus. J. Clin. Diagn. Res..

[36] Pradeep P., Kawatra B., Singh P. (2015). Surgical removal of apically extruded fractured H-File. Guident.

[37] Basatwar H., Kapse B., Nagmode P., Chechare S., Mundhe A., Godge S. (2022). Intentional Replantation—The novel technique for the management of separated instrument in apical third of tooth: A case report. J. Oral Med. Oral Surg. Oral Pathol. Oral Radiol..

[38] Jaiswal S., Sharma A., Gupta S., Sharma S., Sahay S., Tyagi S. (2024). Knowledge, Attitudes, and Practice of Guided Dentistry Among Dental Personnel of North India: A Cross-Sectional Study. Cureus.

[39] Mohanty A., Patro S., Das A., Miglani S., Luke A.M., Pawar A.M., Reda R., Zanza A., Testarelli L. (2023). Nationwide Trends of Modern Endodontic Practices Related to Working Length, Instrumentation, Magnification, and Obturation: A Comparative Cross-Sectional Survey Comparing Endodontic and Non-Endodontic Specialties Practicing Root Canal Treatment in India. J. Multidiscip. Healthc..

[40] Solomonov M., Webber M., Keinan D. (2015). Fractured Endodontic Instrument: A Clinical Dilemma Retrieve, Bypass or Entomb?. J. Mich. Dent. Assoc..

[41] Song Z., Chen B., Liu H., Yi M., Wu M., Wang X. (2020). Instrument Separation Outside the Root Canal: Handling Methods and Difficulties. Dent. Oral Biol. Craniofacial Res..

[42] Richert R., Farges J.-C., Villat C., Valette S., Boisse P., Ducret M. (2021). Decision Support for Removing Fractured Endodontic Instruments: A Patient-Specific Approach. Appl. Sci..

[43] Becker B.D. (2018). Intentional replantation techniques: A critical review. J. Endod..

[44] Setzer F.C., Kratchman S.I. (2022). Present status and future directions: Surgical endodontics. Int. Endod. J..

[45] Abdullah A., Singh N., Rathore M.S., Tandon S., Rajkumar B. (2016). Comparative Evaluation of Electronic Apex Locators and Radiovisiography for Working Length Determination in Primary Teeth in vivo. Int. J. Clin. Pediatr. Dent..

[46] Wesson C.M., Gale T.M. (2003). Molar apicectomy with amalgam root-end filling: Results of a prospective study in two district general hospitals. Br. Dent. J..

[47] Elhakim A., Abd El-Wahab T.M. (2022). A Rare Case of Orthograde Retrieval of Extruded Instrument following Periapical Tissue Healing. Case Rep. Dent..

[48] Li X., Huang L., Li S., Lao S., Yan N., Wu H., Yang X. (2024). Endodontic Microsurgery with the Aid of Dynamic Navigation System Using Minimally Invasive Incision Approach in Anatomically Complex Scenarios: A Case Series. J. Endod..

[49] Palma-Fernandes M.J., Ruiz-Marrara J., de Campos-Muller M.F., de Melchior M.O., Côrte-Real I.S.G., Mazzi-Chaves J.F., Magri L.V. (2025). Teaching Occlusal Splints in the Digital Age: Comparing Student Experiences with Conventional and CAD/CAM Workflows. J. Dent. Educ..

[50] Wei X., Du Y., Zhou X., Yue L., Yu Q., Hou B., Chen Z., Liang J., Chen W., Qiu L. (2023). Expert consensus on digital guided therapy for endodontic diseases. Int. J. Oral Sci..

[51] Liu B., Zhou X., Yue L., Hou B., Yu Q., Fan B., Wei X., Qiu L., Huang Z., Xia W. (2023). Experts consensus on the procedure of dental operative microscope in endodontics and operative dentistry. Int. J. Oral Sci..

[52] Plotino G., Pameijer C.H., Grande N.M., Somma F. (2007). Ultrasonics in Endodontics: A Review of the Literature. J. Endod..

[53] Wang X., Xiao Y., Song W., Ye L., Yang C., Xing Y., Yuan Z. (2023). Clinical application of calcium silicate-based bioceramics in endodontics. J. Transl. Med..

[54] Dioguardi M., Quarta C., Sovereto D., Troiano G., Melillo M., Di Cosola M., Cazzolla A.P., Laino L., Lo Muzio L. (2021). Autotransplantation of the Third Molar: A Therapeutic Alternative to the Rehabilitation of a Missing Tooth: A Scoping Review. Bioengineering.

[55] Peer M. (2004). Intentional replantation—A ‘last resort’ treatment or a conventional treatment procedure? Nine case reports. Dent. Traumatol..

